# Understanding basic and social emotions in Alzheimer's disease and frontotemporal dementia

**DOI:** 10.3389/fpsyg.2025.1535722

**Published:** 2025-02-07

**Authors:** Carlotta Sola, Vanessa Zanelli, Maria Angela Molinari, Claudia Casadio, Francesco Ricci, Omar Carpentiero, Manuela Tondelli, Fausta Lui, Paolo Frigio Nichelli, Francesca Benuzzi

**Affiliations:** ^1^Physical Medicine and Rehabilitation Unit, AUSL-IRCCS di Reggio Emilia, Reggio Emilia, Italy; ^2^Department of Biomedical, Metabolic and Neural Sciences, University of Modena and Reggio Emilia, Modena, Italy; ^3^Azienda Ospedaliero Universitaria (AOU) of Modena, Modena, Italy

**Keywords:** Theory of Mind, emotion recognition, emotional prosody, Alzheimer's disease, frontotemporal dementia

## Abstract

**Introduction:**

Recent developments in the field of social cognition have led to a renewed interest in basic and social emotion recognition in early stages of Alzheimer's Disease (AD) and FrontoTemporal Dementia (FTD). Despite the growing attention to this issue, only few studies have attempted to investigate emotion recognition using both visual and vocal stimuli. In addition, recent studies have presented conflicting findings regarding the extent of impairment in patients in the early stages of these diseases. The present study aims to investigate emotion understanding (both basic and social emotions), using different tasks with visual and auditory stimuli, to identify supramodal deficits in AD and FTD to provide a reliable tool to better outline their behavioral and emotional profile and useful instruments for their management.

**Methods:**

Eighteen patients with AD and 15 patients with FTD were included in the study. Healthy control (HCs) subjects were recruited to obtain normative data for basic emotion recognition tests and social emotion recognition tasks. To evaluate basic emotion recognition, the Facial Emotion Recognition Battery (FERB) and the Emotional Prosody Recognition Battery (EPRB) were administered. To evaluate social emotion recognition, the Faux Pas (FP), Reading the Mind in the Eyes (RME), and Reading the Mind in the Voice (RMV) tests were employed.

**Results:**

FTD patients performed significantly worse than HCs in most of the subtests of the basic emotion recognition batteries, where, instead, AD patients were significantly impaired only when required to match emotional facial expression in different individuals (subtask of the FERB). Moreover, FTD patients scored significantly lower in RME and RMV tests compared both to AD patients and to HCs. In addition, ADs were selectively impaired in RMV as respect to HCs.

**Discussion:**

FTD patients showed deficits in emotion recognition, affecting both basic and social emotions, whether conveyed through facial expressions or prosody. This result may explain the well-known social behavioral difficulties observed in FTD patients from the early stages of the disease. The fewer and specific deficits in AD patients with comparable MMSE scores may be attributed to the mild degree of impairment, as these deficits may appear later in the progression of AD.

## 1 Introduction

The term “Social Cognition” refers to several abilities involved in social information processing, consisting of inferring emotions and socially relevant stimuli to modulate behavior (Adolphs, 1999; Frith, 2008).

Emotional processing plays an important role among high-level social abilities; several studies support the idea that it relies on a broad neural network including fusiform face area (FFA; Kanwisher and Yovel, 2006), amygdala (Adolphs et al., 1998, 1994; Todorov et al., 2013), insula (Wicker et al., 2003; Craig, 2009). Additional areas appear to be specifically involved in prosody, an important social signal, during emotional recognition, including superior temporal sulcus (STS; Grandjean et al., 2005; Sander et al., 2005), middle temporal gyrus (MTG), inferior frontal gyrus (IFG; Johnstone et al., 2006), putamen, pallidum, subthalamic nucleus, and cerebellum (Ceravolo et al., 2021).

Recent data have emphasized the need for a supramodal approach to understanding the neural basis of emotion processing (Schirmer and Adolphs, 2017). Each distinct input channel engages partly non-overlapping neuroanatomical systems with different processing specializations. Then, elaborations of signals across different modalities converge into supramodal representations in areas involving a modality-non-specific abstract code, such as STS, prefrontal and posterior cingulate cortex.

Deficits in emotional processing are observed in both Alzheimer's Disease (AD) and Frontotemporal Dementia (FTD). In AD, mild impairments in emotion recognition, particularly for low-intensity or negative emotions, emerge early and worsen over time (Luzzi et al., 2007; Maki et al., 2013; Torres et al., 2015; Garcia-Cordero et al., 2021; Amlerova et al., 2022; Chaudhary et al., 2022). These deficits extend to multiple sensory modalities, including emotional prosody, likely due to overlap between memory and emotional processing regions affected by neurodegeneration (Bediou et al., 2012). On the other hand, in FTD, significant impairments in recognizing visual and vocal emotional stimuli, especially negative emotions, are more severe than in AD (Fernandez-Duque and Black, 2005; Dara et al., 2013; Bertoux et al., 2015; Bora et al., 2016; Jiskoot et al., 2021; Wright et al., 2018). These deficits, prominent in the behavioral variant (bvFTD), are linked to atrophy in brain regions involved in emotional and social cognition (Rascovsky et al., 2011).

Within social cognition abilities, Theory of Mind (ToM) pertains to the capacity to attribute mental states to others and to anticipate, describe, and elucidate behavior based on these mental states (Baron-Cohen, 1997). Traditionally, ToM is divided in two subcomponents: cognitive ToM and affective ToM (Zhou et al., 2023), which rely on different neural networks. The capacity to understand others' beliefs, intentions and goals (cognitive ToM; Amodio and Frith, 2006) has been connected to the activity of the dorsolateral prefrontal cortex (dlPFC; Shamay-Tsoory and Aharon-Peretz, 2007). On the other hand, the ventromedial prefrontal cortex (vmPFC) and the orbitofrontal cortex (OFC), together with the amygdala, are involved in the representation and top-down regulation of emotional states and represent the node for the affective processing of others' mental states (Abu-Akel and Shamay-Tsoory, 2011).

Whereas, cognitive ToM has been explored using the first-order (Baillargeon et al., 2010) and the second-order (Perner and Wimmer, 1985) false belief tasks, affective ToM has been usually investigated by using the Reading the Mind in the Eyes task (RME; Baron-Cohen et al., 2001) and the Reading the Mind in the Voice task (RMV; Rutherford et al., 2002; Golan et al., 2007). Additionally, the Faux Pas test (Baron-Cohen et al., 1999) is commonly used to assess ToM in a non-specific way.

Despite the extensive research on ToM abilities in neurodegenerative diseases, the findings are highly heterogeneous. Although some studies have shown that AD patients exhibit deficits only in ToM tasks that require high cognitive demand (Castelli et al., 2011; Demichelis et al., 2020; Kessels et al., 2021; de Lucena et al., 2023), other results suggest that certain subcomponents of ToM abilities are preserved in AD (e.g., interpretation of sarcasm, social inference, and emotion evaluation; Kumfor et al., 2017). In contrast, research on patients with FTD has shown more consistent results, with a widespread and severe impairment of ToM abilities, which could serve as a clinical marker distinguishing FTD from other neurodegenerative diseases (Gossink et al., 2018; Dodich et al., 2021).

Given the clinical and social importance of AD and FTD, and the relevance of social cognition in these two neurodegenerative diseases, the purpose of this study was to better characterize them, by using a complete assessment to investigate both visual and auditory processing, both for basic and for social emotions, in the same patients. Although these tests may not reveal such striking differences that can be used for individual diagnosis, we aim to provide a reliable tool to better outline the behavioral and emotional profile of these two pathologies, thus also providing useful instruments for their management. To this aim, we used both visual and prosodic stimuli, specifically, two batteries (the Facial Affect Recognition Battery and the Prosodic Affect Recognition Battery) devised by our research group (Benuzzi et al., 2004; Ariatti et al., 2008). Furthermore, processing of social emotions (affective ToM) within the visual and the prosodic domain was assessed by the RME and RMV tasks. Finally, the Faux Pas test (FP) was used to assess the cognitive component of ToM abilities.

We hypothesized that early stages FTD patients would exhibit a global impairment on emotion recognition tasks and in the affective component of ToM abilities, as opposed to substantially preserved functions in early stages AD patients.

## 2 Materials and methods

### 2.1 Participants

Patients affected by either AD or FTD were recruited among those followed by the Neuropsychology Service of the University Hospital (AOU) of Modena. Eighteen patients with AD (mean age = 72.8 years, SD ± 4.8 years; mean school age = 7.3 years, SD ± 4.5 years; mean MMSE = 25.4, SD ± 3.7) and 15 patients with FTD (mean age = 65.9 years, SD ± 8.7 years; mean school age = 8.4 years, SD ± 4.5 years; mean MMSE = 25.3, SD ± 4.9) were included in the study. They were all right-handed (assessed using the Edinburgh Inventory; Oldfield, 1971) and diagnosed with either AD or FTD according to criteria given by McKhann et al. (2011). Exclusion criteria were as follows: MMSE < 16, history of stroke, history of psychiatric illness or of traumatic brain injury.

As control groups, 70 healthy controls (HC; mean age = 68 years, SD ± 7.7 years; mean school age = 8.5 years, SD ± 3.9 years) were recruited through public announcements among employees or former employees of the University of Modena and Reggio Emilia. They were administered the emotion recognition battery. Among these HC participants, 20 were also submitted to the ToM tasks (mean age = 67.6 years, SD ± 12.7 years; mean school age = 9.4 years, SD ± 4.6 years). HCs were recruited according to the following exclusion criteria: no history of neurological or psychiatric diseases, alcoholism, brain injury, cerebrovascular disease or other neurological conditions. Moreover, exclusion criteria included the presence of depression and obsessive-compulsive disorders, since it has been demonstrated that these disorders interfere with emotion identification (Gur et al., 1992; Abbruzzese et al., 1995; Bouhuys et al., 1999). The presence of these diseases was assessed through the Beck Depression Inventory (BDI > 11; Sica and Ghisi, 2007) and a reduced version of Maudsley Obsessive-Compulsive Questionnaire (MOCQ-R < 75th percentile; Sanavio et al., 1986). See Table 1 for the demographic and clinical features of groups (AD, FTD, HC).

**Table 1:** Demographic and clinical data of the study population.

	**Age (years)**		**Education level (years)**		**Gender**		**MMSE (corrected)**	
	***M***	**SD**	***M***	**SD**	**Male**	**Female**	***M***	**SD**
AD	72.8	4.8	7.3	4.5	6	12	25.4	3.7
FTD	65.9	8.7	8.4	4.5	11	4	25.3	4.9
HC (emotion recognition battery)	68	7.7	8.5	3.9	23	46		
HC (ToM tasks)	67.6	12.7	9.4	4.6	7	13		

AD, Alzheimer's Disease; FTD, FrontoTemporal Dementia; HC, Healthy Controls; M, mean; SD, standard deviation; MMSE, Mini Mental State Examination.

All subjects gave their informed consent to participate in the study. Consent was obtained according to the Declaration of Helsinki. Moreover, the study procedure was approved by the ethical committee of the University of Modena and Reggio Emilia (Comitato Etico di Ateneo per la Ricerca, CEAR; Prot. n 83243). Both patients and HC underwent a clinical neuropsychological evaluation which was conducted during a single session lasting ~1 h and a half. On a subsequent day, experimental tests (emotion recognition batteries and ToM tasks) were administered in a single session lasting between 40 min and 60 min, depending on the individual patients' abilities.

### 2.2 Materials

#### 2.2.1 Basic emotion recognition

To evaluate the ability to process basic emotion (fear, happiness, sadness, anger, and disgust; Ekman, 1999), two batteries were used (Benuzzi et al., 2004).

The Facial Emotion Recognition Battery includes the following subtests:

Face Matching (FM). In this task, subjects are presented with a vertically arranged set of four neutral expression faces and must select the photograph identical to a target face. Photographs of different individuals of the same gender are used as distractors. The task includes 14 trials and assesses perceptual deficits in face discrimination, for each correct answer, one point was assigned (range score 0–14), thus the higher the score, the better the performance.

Facial Identity Recognition (FIR). This task evaluates the ability to recognize a single person across various facial expressions. It consists of 14 trials, in which the subject is asked to identify the target person from a vertically arranged set of four faces, each showing different expressions. The task assesses associative deficits in face perception. For each correct answer, one point was assigned (range score 0–14), thus the higher the score, the better the performance.

Facial Affect Naming (FAN). Subjects are asked to choose the name that best describes the emotional expression displayed from five options printed below a stimulus face. The subtest includes 25 trials, with five trials for each basic emotion, for each correct answer, one point was assigned (range score 0–25), thus the higher the score, the better the performance.

Facial Affect Selection (FAS). The participant is asked to select the face with an expression that matches a target label from a vertically arranged set of five ones. The test includes 25 trials, for each correct answer, one point was assigned (range score 0–25), thus the higher the score, the better the performance.

Facial Affect Matching (FAM). In this task, subjects must choose from a vertically arranged set of five faces, the one displaying the same expression as a stimulus face. The person in the stimulus photo is always different, with one identity foil included, i.e., a photograph of the same individual as the stimulus, but with a different expression. The test comprises 25 trials, for each correct answer, one point was assigned (range score 0–25), thus the higher the score, the better the performance.

FM and FIR subtests represent control tasks, since they assess the ability to discriminate the perceptual features of faces. On the other hand, FAN, FAS, and FAM assess basic emotion processing and recognition. There is no time limit to complete the task.

The Emotional Prosody Recognition Battery (Benuzzi et al., 2004; Ariatti et al., 2008; Bonora et al., 2011) evaluates the ability to process basic emotions from prosodic cues presented via a computer application. Before the administration of the battery, all participants underwent an auditory acuity evaluation. All subjects (HC, AD, and FTD) had a normal hearing threshold. Stimuli are brief Italian sentences with a neutral meaning (e.g., “Marta is combing the cat”). Sentences vary only with respect to emotional prosody, which could express one of the five basic emotions: fear, anger, sadness, happiness, and disgust. At the beginning of the testing session, the computer volume is regulated by the examiner according to the subject's requests. Sentences are presented both orally and in written form on a computer screen at the same time, and subjects can listen to each trial up to three times. The Emotional Prosody Recognition Battery includes different subtests as follows:

Vocal Identity Discrimination (VID) assesses basic voice discrimination abilities. Participants are asked to determine whether two sentences are spoken by the same person. VID consists of 16 pairs of neutral (aprosodic) stimuli, for each correct answer, one point was assigned (range score 0–16), thus the higher the score, the better the performance.

Prosodic Discrimination (PrD) measures basic intonation discrimination abilities. Given two sentences, subjects must identify whether they are uttered with the same prosodic intonation. PrD consists of 16 pairs of sentences expressing four different intonations: interrogative, declaratory, exclamatory, and imperative, for each correct answer, one point was assigned (range score 0–25), thus the higher the score, the better the performance.

Prosodic Affect Naming (PrAN) assesses emotional prosodic recognition abilities. Subjects are asked to choose from five options on the screen (representing five basic emotions) the one that best describes the emotional prosody of the target recorded sentence. PrAN consists of 25 trials, for each correct answer, one point was assigned (range score 0–25), thus the higher the score, the better the performance.

Prosodic Affect Discrimination (PrAD) measures emotional prosodic discrimination abilities. Given two recorded sentences, subjects must decide whether they are spoken with the same emotional prosody. PrAD consists of 45 pairs of sentences expressing the five basic emotions, for each correct answer, one point was assigned (range score 0–45), thus the higher the score, the better the performance.

Similarly to what happens for the Facial Emotion Recognition Battery, some sub-tests (here, VID, and PrD) represent control tasks, since they assess basic prosodic recognition abilities. On the contrary, PrAN and PrAD assess the emotional prosodic discrimination ability. There is no time limit for answering.

#### 2.2.2 Social emotion recognition (ToM tasks)

To assess social emotion recognition, the following three tasks were used: the Reading the Mind in the Eyes test (RME; Baron-Cohen et al., 2001), the Reading the Mind in the Voice (RMV; Rutherford et al., 2002; Golan et al., 2007) and the Faux Pas test (FP; Stone et al., 2002).

For the RME test (Baron-Cohen et al., 2001), we translated the official version of the Baron-Cohen test (https://www.autismresearchcentre.com/tests/eyes-test-adult/) into Italian, since the first participants were tested before the Italian adaptation was published. Furthermore, we selected 30 out of the original 36 items, excluding items where the verbal label in Italian corresponded to a word with very low usage frequency. In order to ensure methodological consistency, data collection was carried out in the same manner for all the subsequent participants. An independent group of 15 healthy subjects validated the chosen stimuli. The selected 30 images were presented using PowerPoint on a 15″ screen. Each slide featured a black-and-white photograph of the eye region of a human face against a white background, accompanied by four adjectives (e.g., bothered, joking, passionate, comforting). Subjects are asked to choose the adjective that best describes the mental state expressed by the person in the image. There was no time limit for responding.

RMV (Rutherford et al., 2002; Golan et al., 2007) assesses the ability to recognize one's intention through the prosody. We adapted the original version of the task to Italian, including two different distractors for each trial (see below), selecting 35 new short sentences that were recorded by means of the Audacity software 1.2.6 (http://audacity.sourceforge.net/). Then, these sentences were validated by an independent group of healthy subjects and 30 of them were selected for the task.

Stimuli are presented both orally and in written form at the same time, on a computer screen through Microsoft Office PowerPoint. The items were designed so that the meaning of each sentence never matched the prosody with which it was pronounced. For instance, the sentence “I swear I have” typically indicates the completion of an action. However, when pronounced with a sarcastic tone, it implies the opposite, namely that the action has not been completed. The task required subjects to listen to the sentence and select the label that best describes the prosodic meaning conveyed by the sentence. The labels include: (i) an adjective that accurately reflects the prosody (correct answer); (ii) an adjective that corresponds to the sentence's semantic meaning (semantic error); (iii) an adjective that matches neither the prosody nor the meaning of the sentence (incorrect answer).

FP (Stone et al., 2002) assesses both the cognitive and affective components of ToM. Given the length of time required to administer the entire test, the 10 least complex stories in terms of comprehension were selected, choosing five stories that contain faux pas and five stories that do not (control tasks), from the Italian version developed by Massaro and colleagues (https://www.autismresearchcentre.com/tests/faux-pas-test-adult). The task requires participants to listen to a story read by an examiner. Some stories contain a faux pas, in which a character says or does something that unintentionally offends or embarrasses another character, while others do not. After each story, participants are asked to answer a series of questions designed to test their understanding of the social dynamics. These questions include: (i) Faux Pas detection; (ii) Theory of Mind questions; and (iii) Control questions to ensure basic comprehension. The total score was obtained from the sum of the single scores. Correct identification of faux pas and correct answers to ToM-related questions indicate an understanding of social nuances and the ability to infer others' mental states, while errors might indicate difficulties in recognizing or interpreting social information.

#### 2.2.3 Neuropsychological assessment

All patients were submitted to a comprehensive neuropsychological evaluation. For the purpose of the present study, performances in the following tasks were considered: Mini-Mental State Examination (MMSE; Magni et al., 1996) for the assessment of the stage of the neurodegenerative disease, Benton test of facial recognition (Benton et al., 1983) to identify the presence of perceptual difficulties in processing faces and the Similarities subtest from the Italian revised version of the Wechsler Adult Intelligence Scale (Orsini and Laicardi, 1997), as a measure of executive functions.

### 2.3 Statistical analyses

Data management and analysis were performed using RStudio (2024; RStudio: Integrated Development for R. RStudio, PBC, Boston, MA URL http://www.rstudio.com/) and Statistica (https://docs.tibco.com/).

Normality of variables was assessed using the Shapiro-Wilk test.

Parametric tests (repeated-measures ANOVAs; *t*-test for independent samples) and non-parametric tests (Kruskal-Wallis rank sum test; Pearson's Chi-Squared test) were used to investigate differences among groups. For *post-hoc* comparisons, Newman-Keuls tests were used and they were adjusted by Bonferroni corrections for multiple comparisons to account for the probability of committing type-1 errors. Finally, Pearson or Spearman's correlations were performed to assess the relationship between neuropsychological scores and emotion recognition test scores.

## 3 Results

Analyses of data distribution using the Shapiro-Wilk test showed that age and corrected MMSE scores were normally distributed, whereas education and gender distribution were not normal. Analyses of data distribution using the Shapiro-Wilk test showed that basic emotion recognition batteries, RME, RMV, Benton test of facial recognition and Similarities WAIS subtest were normally distributed, whereas FP and MMSE were not.

### 3.1 Demographic data

The one-way ANOVA was conducted with Age as within-subjects factor and Group (AD, FTD, HC) as between-subjects factor. The main effects of the Groups were not significant [*F*_(3, 119)_ = 2.11, *p* = 0.1, η^2^*p* = 0.05]. The Kruskal-Wallis ANOVA by Ranks sum test was conducted with Education as within-subject factor and Group (AD, FTD, HC) as between-subjects factor. The main effect of the Group was not significant (H_3_ = 3.81, *p* = 0.3). The *T*-test for independent samples conducted on corrected MMSE scores between AD and FTD was not significant [*t*_(31)_ = 0.06, *p* = 0.95]. The Chi-squared test of independence was performed to analyze the distribution of gender across the groups (AD, FTD, HC). The test revealed a significant difference in gender distribution among the groups (χ^2^ = 8.46, df = 3, *p*-value = 0.04).

### 3.2 Basic emotion recognition batteries

#### 3.2.1 Facial emotion recognition battery

**Figure 1:**
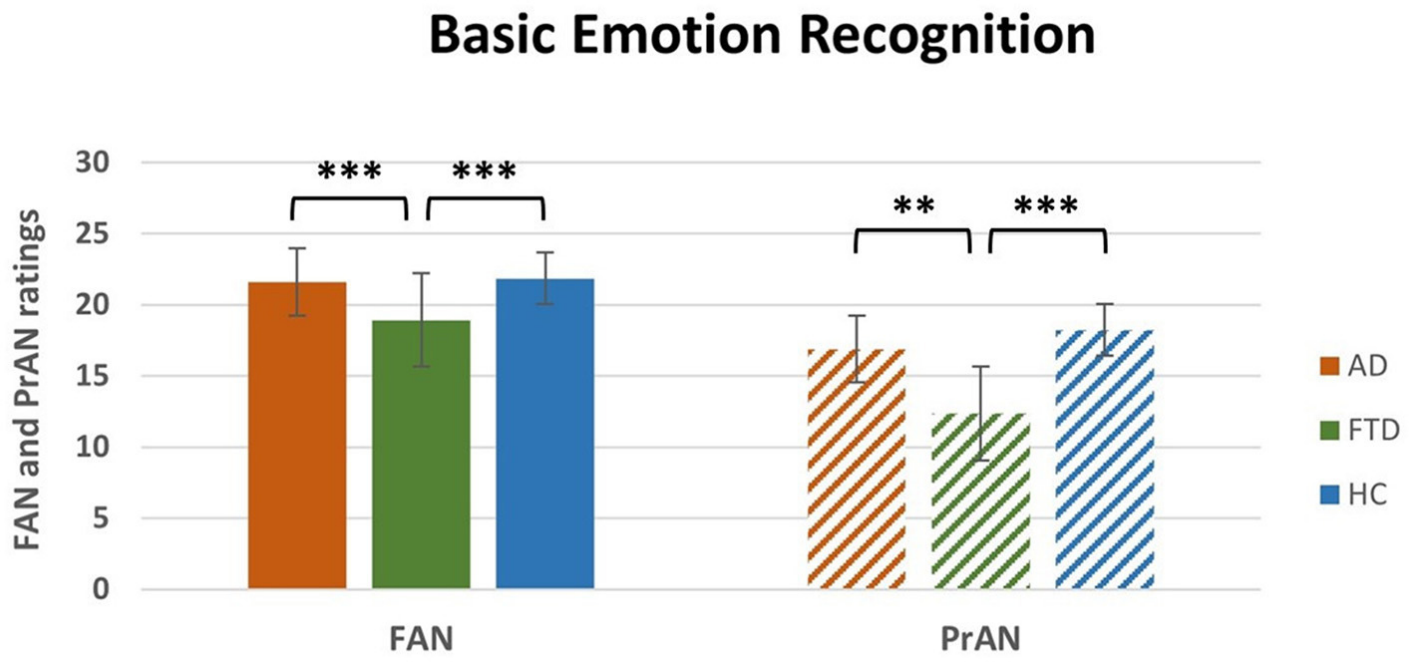
Interaction Subtest * Group *post-hoc* results for FAN and PrAN subtests. FAN, Facial Affect Naming; PrAN, Prosodic Affect Naming; AD, Alzheimer's Disease; FTD, FrontoTemporal Dementia; HC, Healthy Controls. **p < 0.01, ***p < 0.001.

A repeated measures ANOVA was conducted on the Facial Emotion Recognition Battery scores with Subtests (FM, FIR, FAN, FAS, and FAM) as within-subjects factor and Group (AD, FTD, HC) as between-subjects factor (Table 2). The main effects of Group [*F*_(2, 100)_ = 9.4, *p* < 0.001, η^2^*p* = 0.16] and Subtests [*F*_(4, 400)_ = 9.4, *p* < 0.001, η^2^*p* = 0.78] were statistically significant, as well as the interaction Subtests ^*^ Group [*F*_(8, 400)_ = 9.6, *p* < 0.001, η^2^*p* = 0.16]. The *post-hoc* comparisons showed (Table 2) that FTD patients significantly differed in the FAN (*M* = 18.9, SEM = 0.6; Figure 1, left) and in the FAM (*M* = 15.8, SEM = 1.4) subtests from both AD (FAN *M* = 21.6, SEM = 0.1, *p* < 0.01; FAM *M* = 18.7, SEM = 0.2, *p* < 0.001) and HC (FAN *M* = 21.8, SEM = 0.3, *p* < 0.01; FAM *M* = 21.4, SEM = 0.4, *p* < 0.001); in addition, AD differed from HC in the FAM subtest (*p* < 0.01). Interestingly, the significant differences resisted also when conducting an Analysis of Covariance (ANCOVA) considering age and education as covariates [*F*_(2, 98)_ = 14.2, *p* < 0.001]. All the p's resisted Bonferroni's correction.

**Table 2:** Descriptive statistics, repeated measures ANOVA and *post-hoc* results conducted on facial emotion recognition battery (FERB) scores.

**Descriptives**				**ANOVA**			
	**AD (*****n*** = **18)**	**FTD (*****n*** = **15)**	**HC (*****n*** = **70)**	**Group F**	**FERB F**	**Group** × **FERB F**	**Neuman Keuls** ***post-hoc*** **(Cohen's** ***d*****)**
				**9.37** ^***^	**353.74** ^***^	**9.56** ^***^	
**FM**							
*M*	13.61	13.67	13.77				
(sd)	(0.85)	(0.62)	(0.68)				
**FiR**							
*M*	11.83	11.93	12.66				
(sd)	(1.65)	(2.46)	(2.09)				
**FAN**							
*M*	21.61	18.93	21.84				FTD < AD (1.18)^***^ FTD < HC (1.19)^***^
(sd)	(2.28)	(2.25)	(2.64)				
**FAS**							
*M*	21.83	20.47	22.57				
(sd)	(2.31)	(3.25)	(2.53)				
**FAM**							
*M*	18.72	15.8	21.36				FTD < AD (0.6)^***^; AD < HC (1.14)^***^; FTD < HC (1.24)^***^
(sd)	(4)	(5.53)	(3.11)				

AD, Alzheimer Disease; FTD, Frontotemporal Dementia; HC, Healthy Controls; FM, Face Matching; FAN, Facial Affect Naming; FAS, Facial Affect Selection; FAM, Facial Affect Matching; M, mean; sd, standard deviation;

*** p < 0.001.

#### 3.2.2 Emotional prosody recognition battery

A repeated measures ANOVA was conducted on the Emotional Prosody Recognition Battery scores with Subtests (VID, PrD, PrAN, PrAD) as within-subjects factor and Group (AD, FTD, HC) as between-subjects factor (Table 3). The main effects of Group [*F*_(2, 100)_ = 13, *p* < 0.001, η^2^*p* = 0.21] and of Subtests [*F*_(3, 300)_ = 1063.3, *p* < 0.001, η^2^*p* = 0.91] were significant. The interaction Subtests ^*^ Group was also significant [*F*_(6, 300)_ = 4.3, *p* < 0.001, η^2^*p* = 0.08]. The *post-hoc* analyses (Table 3) showed that FTD significantly differed in the PrAN (*M* = 12.3, SEM = 1.2; Figure 1, right) subtest from both AD (*M* = 16.9, SEM = 0.2, *p* < 0.01) and HC (*M* = 18.2, SEM = 0.5, *p* < 0.001). FTD patients' scores (*M* = 33.6, SEM = 0.9) were also significantly different from HC (*M* = 37.7, SEM = 0.6, *p* < 0.001) and AD (*M* = 35.8, SEM = 0.2, *p* = 0.02) in the PrAD subtest. The difference between FTD and AD did not resist Bonferroni's correction for multiple comparisons. All other p's resisted Bonferroni's correction. Interestingly, the significant differences resisted also when conducting an ANCOVA analysis considering age and education as covariates [*F*_(2, 98)_ = 14.7, *p* < 0.001].

**Table 3:** Descriptive statistics, repeated measures ANOVA and *post-hoc* results conducted on emotional prosody recognition battery (EPRB) scores.

**Descriptives**				**ANOVA**			
	**AD (*****n*** = **18)**	**FTD (*****n*** = **15)**	**HC (*****n*** = **70)**	**Group F**	**EPRB F**	**Group** × **EPRB F**	**Neuman Keuls** ***post-hoc*** **(Cohen's** ***d*****)**
				**12.99** ^***^	**1063.32** ^***^	**4.26** ^***^	
**VID**							
*M*	12.17	10.87	12.01				
(sd)	(2.23)	(1.85)	(1.91)				
**PrD**							
*M*	13.06	11.87	13.41				
(sd)	(1.47)	(1.92)	(1.95)				
**PrAN**							
*M*	16.89	12.33	18.21				FTD < AD (1.14)^***^ FTD < HC (1.33)^***^
(sd)	(3.20)	(4.65)	(4.18)				
**PrAD**							
*M*	35.83	33.60	37.69				FTD < HC (1)^***^ FTD < AD (0.7)^*^
(sd)	(3.07)	(3.33)	(4.75)				

AD, Alzheimer Disease; FTD, Frontotemporal Dementia; HC, Healthy Controls; VID, Vocal Identity Discrimination; PrD, Prosodic Discrimination; PrAN, Prosodic Affect Naming; PrAD, Prosodic Affect Discrimination; M, mean; sd, standard deviation;

* p < 0.05;

*** p < 0.001.

### 3.3 Social emotion recognition (ToM tasks)

**Figure 2:**
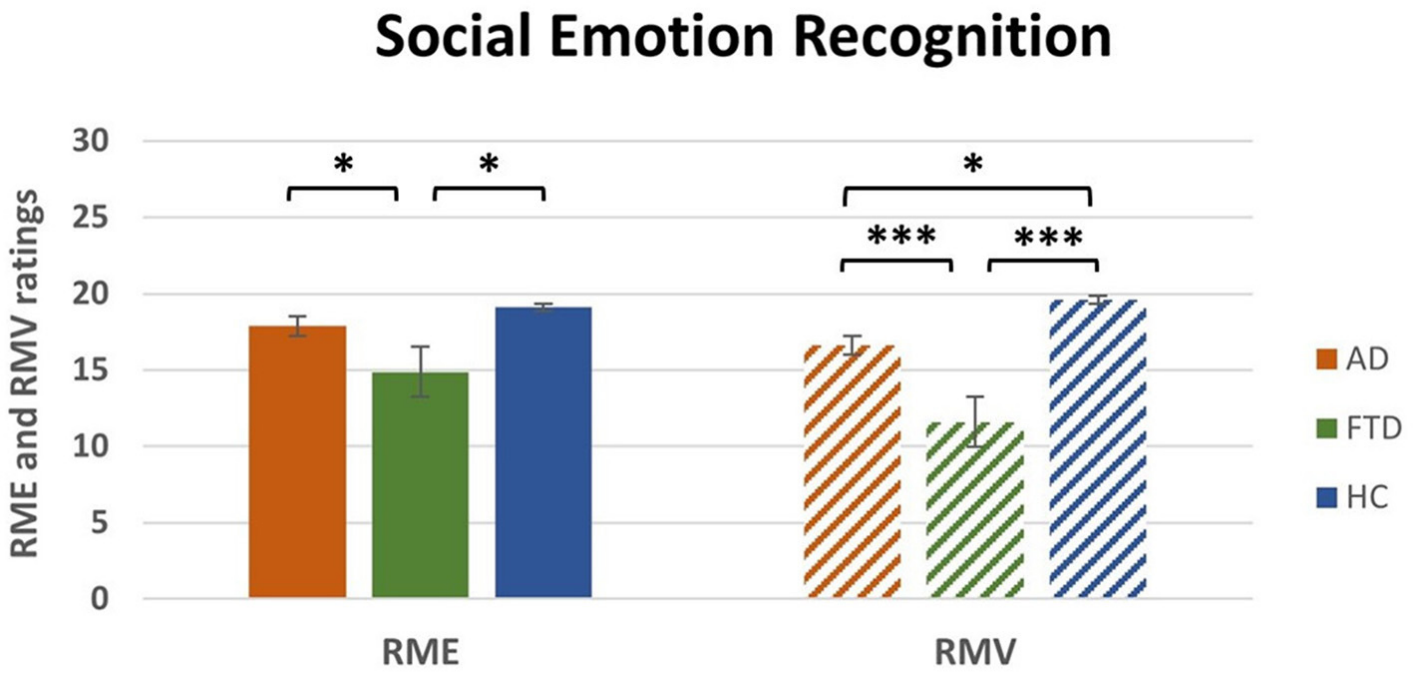
Main effect of group *post-hoc* results for RME and RMV tasks. RME, Reading the Mind from the Eyes; RMV, Reading the Mind in the Voice; AD, Alzheimer's Disease; FTD, FrontoTemporal Dementia; HC, Healthy Controls. *p < 0.05, ***p < 0.001.

One-way between-subject ANOVA on RME ratings revealed a significant effect of Group [*F*_(2, 50)_ = 4.2, *p* < 0.05; η^2^*p* = 0.14, Table 4]. *Post-hoc* comparisons (Table 4) that FTD ratings (*M* = 14.9, SEM = 1) were significantly lower compared to HC (*M* = 19.1, SEM = 1.1; *p* < 0.05) and to AD (*M* = 17.9, SEM = 1; *p* < 0.05). There was no significant difference between HC and AD (Figure 2, left).

**Table 4:** Descriptive statistics, repeated measures ANOVA and *post-hoc* results conducted on ToM scores.

**Descriptives**				**ANOVA**	
	**AD (*****n*** = **18)**	**FTD (*****n*** = **15)**	**HC (*****n*** = **20)**	**Group F**	**Neuman Keuls** ***post-hoc*** **(Cohen's** ***d*****)**
**RMV**					
*M*	16.61	11.6	19.6		FTD < AD (1.47)^***^; HC < FTD (1.84)^***^; HC < AD (0.75)^**^
(sd)	(2.97)	(3.79)	(4.83)	17.329^***^	
**RME**					
*M*	17.89	14.87	19.1		FTD < AD (0.77)^*^ HC < FTD (0.94)^*^
(sd)	(4.06)	(3.81)	(4.95)	4.159^*^	
**RMV-SE**					
*M*	9	11.2	6.6		FTD < AD (0.91)^*^; HC < FTD (1.53)^***^; HC < AD (0.75)^*^
(sd)	(2.66)	(2.14)	(3.68)	10.400^***^	
				**Group H (KW)**	
**FP**					
*M*	44.39	39.27	54.9		HC < FTD (1.65)^***^ HC < AD (1.16)^***^
(sd)	(11.45)	(12.19)	(5.67)	19.1^***^	

RMV, Reading the Mind from the Voice; RME, Reading the Mind from the Eyes; RMV-SE, Reading the Mind from the Voice-Semantic Errors; FP, Faux Pas; KW, Kruskal-Wallis;

* p < 0.05;

** p < 0.01;

*** p < 0.001.

One-way between-subject ANOVA on Semantic Errors of RMV revealed a significant effect of Group [*F*_(2, 50)_ = 10.4, *p* < 0.001; η^2^*p* = 0.29, Table 4]. *Post-hoc* comparisons (Table 4) revealed that FTD ratings (*M* = 11.2, SEM = 0.6) were significantly higher compared to HC (*M* = 6.6, SEM = 0.8; *p* < 0.001) and AD (*M* = 9, SEM = 0.6; *p* < 0.05). Moreover, there was a significant difference between AD and HC (*p* < 0.05).

One-way between-subject ANOVA on RMV ratings revealed a significant effect of Group [*F*_(2, 50)_ = 17.3, *p* < 0.0001; η^2^*p* = 0.41, Table 4]. *Post-hoc* comparisons (Table 4) revealed that FTD ratings (*M* = 11.6, SEM = 1) were significantly lower compared to HC (*M* = 19.6, SEM = 1.1; *p* < 0.001) and to AD (*M* = 16.6, SEM = 0.7; *p* < 0.001). Moreover, there was a significant difference between AD and HC (*p* < 0.05; Figure 2, right).

**Figure 3:**
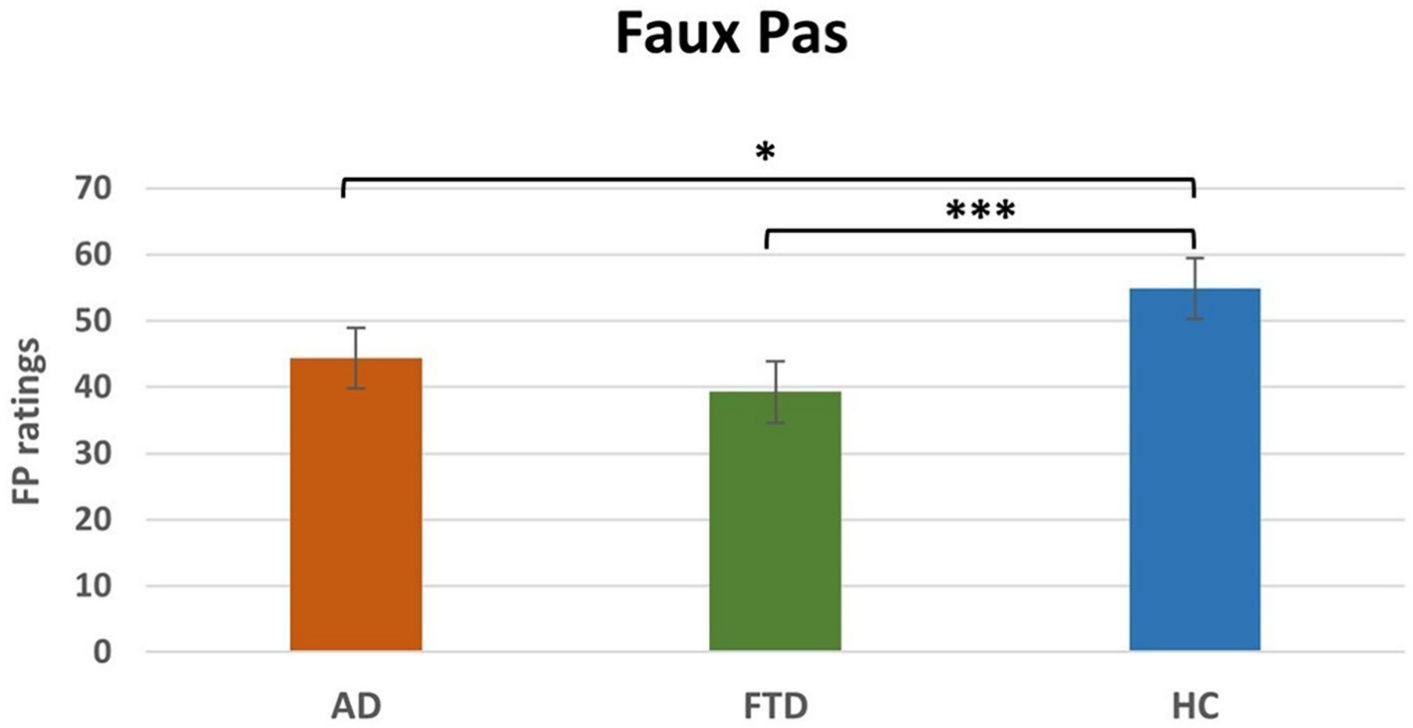
Main effect of group *post-hoc* results for FP. FP, Faux Pas; AD. Alzheimer's disease; FTD, FrontoTemporal Dementia; HC, Healthy Controls. *p < 0.05, ***p < 0.001.

Non-parametric one-way between-subject ANOVA (Kruskal-Wallis rank sum test) on FP ratings revealed a significant effect of Group (H_2_ = 19.1, *p* < 0.0001, Table 4). *Post-hoc* comparisons (Table 4) revealed that FTD (*M* = 16.9, SEM = 3.1; *p* < 0.001) and AD (*M* = 22.6, SEM = 2.7; *p* < 0.05) ratings were significantly lower compared to HC (*M* = 38.5, SEM = 1.3). The difference between FTD and HC resisted Bonferroni's correction, whereas there was no significant difference between AD and FTD (Figure 3).

### 3.4 Neuropsychological tests

There was no significant difference between AD and FTD at the Benton test of facial recognition [*t*_(30.54)_ = 1.38, *p* = 0.18]. The mean score of Similarities WAIS subtest of FTD patients (*M* = 27.32, SEM = 3.8) was significantly different compared to the mean score of AD patients [*M* = 39.91, SEM = 1.5; *t*_(18.51)_ = 3.06, *p* < 0.01].

### 3.5 Correlations

**Figure 4:**
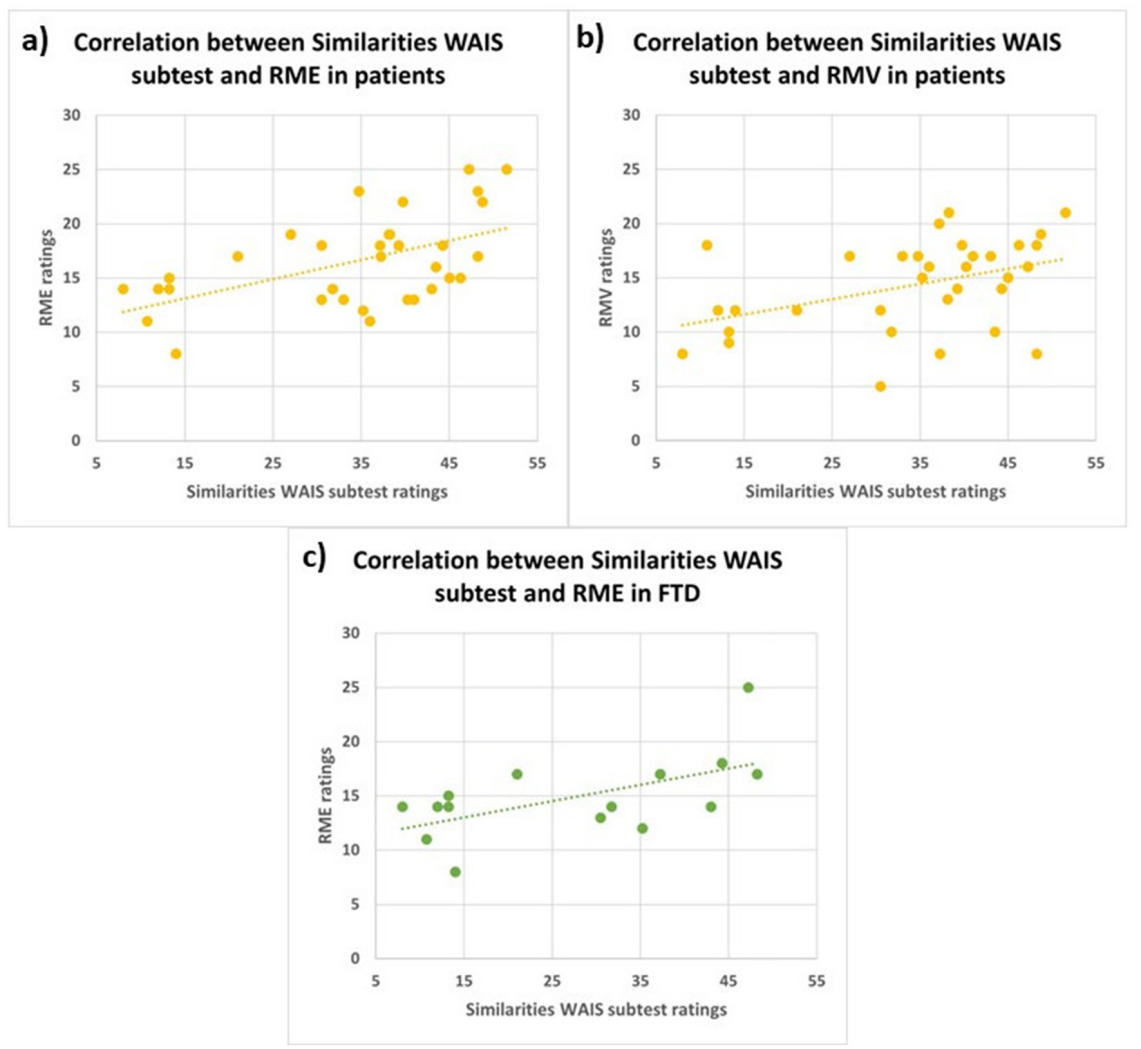
Correlations between similarities WAIS subtest and RME in all patients (**A**; *r* = 0.5, *p* < 0.01), between Similarities WAIS subtest and RMV in all patients (**B**; *r* = 0.4, *p* < 0.05) and between Similarities WAIS subtest and RME in FTD patients (**C**; *r* = 0.6, *p* < 0.05). RME, Reading the Mind in the Eyes; RMV, Reading the Mind in the Voice; FTD, FrontoTemporal Dementia.

Pearson's correlation analyses revealed three significant positive correlations: between Similarities WAIS subtest Test and RME in FTD patients (*r* = 0.6, *p* < 0.05); between Similarities WAIS subtest and RME in all patients (*r* = 0.5, *p* < 0.01); and between Similarities WAIS subtest and RMV in patients (*r* = 0.4, *p* < 0.05; Figure 4).

## 4 Discussion

In the present study we aimed to assess the social skills in the early stages of Alzheimer's Disease (AD) and FrontoTemporal Dementia (FTD), this information can be useful both for a better characterization and for a better clinical management of the two conditions. To this end, we recruited two groups of patients, one with AD and one with FTD, whose cognitive impairment was comparable (MMSE not significantly different between the two groups), and a control group of healthy participants. Both visual and auditory tasks were administered, both for basic emotion and for social emotion (ToM) recognition. In addition, to test cognitive ToM, the Faux Pas (FP) test was used. Overall, our results suggest that in early stages of FTD and AD there is an impairment of social cognition.

Regarding FTD, in line with the literature, our data show that these patients are significantly impaired, as compared to AD patients and HC, in all tasks that evaluate basic and social emotions processing. Specifically, FTD patients' deficits emerge, in comparison to AD patients, in those subtests of Facial Emotion Recognition Battery and Emotional Prosody Recognition Battery which require the association of an emotional (visual and auditory) expression with a verbal label, that is, in the Facial Affect Naming (FAN) and Prosodic Affect Naming (PrAN) subtests. Various studies showed consistent deficits in emotion recognition common to both visual (Bertoux et al., 2015; Jiskoot et al., 2021) and vocal stimuli (Dara et al., 2013; Wright et al., 2018), and particularly severe for negative emotions (Rosen et al., 2002; Fernandez-Duque and Black, 2005). Indeed, social cognition deficits are widely recognized as a hallmark of FTD, especially in the behavioral variant (bvFTD; Rascovsky et al., 2011). Notably, this impairment is particularly relevant in bvFTD as well as in the semantic variant of FTD (svFTD; Kumfor et al., 2014; Lee et al., 2020), and has been linked to structural atrophy in brain regions such as the anterior temporal lobes and the amygdala (Rosen et al., 2002, 2006; Kumfor et al., 2014; Lee et al., 2020). On the other hand, previous findings showed that tasks that increase the intensity of emotional expressions may mitigate recognition issues in bvFTD and primary progressive non-fluent aphasia (PNFA), suggesting that attentional and perceptual difficulties contribute to deficits in some FTD subtypes (Rascovsky et al., 2011). However, in svFTD, these issues are likely due to primary emotion processing impairments, rather than to cognitive overload. Interestingly, negative emotion recognition turned out to be particularly useful for differentiating FTD from AD (Bora et al., 2016), as AD patients typically show milder deficits.

Deficits in the ToM skills are usually found in FTD patients, especially in the behavioral variant, with respect to AD patients, both in visual (Gregory et al., 2002) and auditory modality (Orjuela-Rojas et al., 2021). Our findings demonstrate that FTD patients exhibit significantly greater impairments in both RME and RMV tests, compared to HC. Additionally, FTD patients show significantly worse performance in the RMV test compared to AD patients. Interestingly, especially in FTD patients, we found that Similarities WAIS subtest and RME scores positively correlated, showing that the higher the deficit in executive functions, the higher the impairment in recognizing emotions from eyes' cues. The association between executive functions and emotional recognition has been reported in healthy aging (Circelli et al., 2013) and in several psychiatric diseases (David et al., 2014; Yang et al., 2015; Williams et al., 2015), as well as in neurodegenerative diseases, such as Parkinson's Disease (Péron et al., 2012) and AD (Buçgün et al., 2023). In particular, social cognition skills could rely on executive processes, such as mental speed, cognitive flexibility, and inhibitory control to disregard personal viewpoints and concentrate on pertinent aspects, enabling the timely processing of all relevant information (David et al., 2014). Indeed, the ability to understand others' beliefs, intentions, and goals (cognitive ToM) relies on a frontotemporal network comprising the dorsolateral prefrontal cortex (dlPFC; cognitive processing of mental states and perspective-taking), the ventromedial prefrontal cortex (vmPFC), the orbitofrontal cortex (OFC), and the amygdala (processing and regulating emotional states; Amodio and Frith, 2006; Shamay-Tsoory and Aharon-Peretz, 2007; Abu-Akel and Shamay-Tsoory, 2011). On the other hand, the posterior superior temporal sulcus (pSTS), the temporo-parietal junction (TPJ), and medial prefrontal cortex (mPFC) belong to both emotional and cognitive ToM networks (Schurz and Perner, 2015; Molenberghs et al., 2016). This network facilitates representing others' mental states and differentiating them from one's own, regardless of the nature of the states.

Lastly, we found that FTD patients in the RMV test made a significant amount of semantic errors as compared to healthy controls. This indicates that these patients were able to understand the sentences' meaning, nevertheless they exhibited selective impairment in recognizing the affective aspects of prosody.

Focusing on AD, our study revealed a clear impairment in the recognition of emotional expression in the Facial Affect Matching (FAM) subtest, which requires to keep in memory and compare two emotional stimuli. This deficit could be explained by the overlap between areas engaged in memory tasks, and those involved in emotional processing, both prone to neurodegeneration in AD (Bediou et al., 2012). Indeed, most of the previous studies concluded that the ability to understand facial and prosodic emotional expressions is likely impaired because of the general cognitive decline observed in these patients (Amlerova et al., 2022; Buçgün et al., 2023). The mild deficits described in emotion recognition in the early stages of AD were more specifically related to low-intensity or negative emotions, such as sadness (Maki et al., 2013; Torres et al., 2015; Garcia-Cordero et al., 2021). On the other hand, emotion recognition seemed preserved in tasks with low cognitive demand (Luzzi et al., 2007). Furthermore, deficits in emotional processing in AD also extended across different sensory modalities (e.g., prosody; Amlerova et al., 2022). Therefore, the deficits observed in AD patients in the FAM subtest could be related to the high cognitive demand intrinsic to the task, since the patient is required to remember a face and associate it with an emotional label.

Regarding the ToM skills in AD patients, according to Wright et al. (2018), the recognition of affective prosody relies on a ventral processing stream involving the superior temporal cortex as well as the inferior and anterior temporal cortex in the right hemisphere. Impairments in this pathway may result in a compromised access to the Abstract Representations of Acoustic Characteristics that Convey Emotion (ARACCE; Wright et al., 2018). This is in line with our findings that AD patients are selectively impaired in recognizing emotions from the voice, that is, from the prosody, as compared to the HC group. These emotional recognition deficits in AD are consistent with neurodegeneration in temporal lobes (Bediou et al., 2012; Amlerova et al., 2022), affecting the abstract representations of acoustic features that convey emotions.

Finally, FTD and AD patients are significantly impaired in several Faux Pas (FP) subtests, including the affective and cognitive scores, as compared to the HC, whereas the groups of patients did not differ from each other. Our FP task contained several questions which enabled us to assess whether patients understood both the semantic aspects of the stories (control stories and questions) and the social gaffes (faux pas stories and questions).

Recent neuroimaging studies showed that the areas associated with the RME task are the left and right middle temporal gyri, superior temporal gyrus, cingulate gyrus, superior frontal gyrus, inferior frontal gyrus, middle frontal gyrus and left precentral gyrus. A recent FDG-PET and MRI (Magnetic Resonance Imaging) study hypothesizes that the ToM neural correlates can be categorized into hubs and spokes (Orso et al., 2022). Within the connectionist paradigm (van den Heuvel et al., 2009), it has been suggested that regions with greater connectivity to other components of a network (i.e., the “hubs”) play a more crucial role in network functioning than those with less connectivity (i.e., the “spokes;” Hwang et al., 2013). Moreover, it has been hypothesized that damage to secondary nodes (spokes) can be compensated by the integrity of central nodes (hubs), whereas damage to the hubs themselves may result in clinical symptoms (van den Heuvel et al., 2009; Hwang et al., 2013). According to the structural connectivity and distribution of hypometabolism, hubs of the RME network were identified in frontal regions. This may explain ToM deficits commonly observed in FTD patients, where neurodegeneration impacts these hubs in the early stages of the disease (Adenzato et al., 2010). In contrast, in AD, their functional involvement typically becomes evident in the later stages of the disease, thus explaining the absence of ToM impairments in the early stages of the disease (Lucena et al., 2020). Indeed, our results in the RME subtest are consistent with these hypotheses, in that we only found deficits in FTD patients, which could likely be due to the neurodegeneration of these hubs.

To the best of our knowledge, this is the first extensive evaluation of emotional and social abilities in groups of neurodegenerative patients in the Italian population. Namely, we revised the RMV task to better assess the prosodic affective component of ToM in FTD patients. In particular, we introduced the possibility to evaluate semantic vs. non-semantic errors. The development of two tools for studying ToM abilities in the Italian language fills a gap in neuropsychological testing by providing instruments specifically adapted for use with Italian patients, which were previously unavailable. Furthermore, alongside the ability to quantify errors in the RMV test, we have introduced the capability to qualify these errors. This allows for the identification of patients with deficits in mental state processing stemming from semantic impairments, as opposed to those whose errors may be attributed to task complexity, thus reflecting the underlying neurodegenerative process. Thus, the modified versions of the two ToM tests are sensitive to detecting deficits that cannot be attributed to a generic neurodegenerative process. Combined with the two emotion processing batteries, they represent effective tools for both the quantification and qualification of social cognition impairments, even in patients with different neurological conditions such as traumatic brain injury, epilepsy (as demonstrated in our previous studies; Benuzzi et al., 2004; Bonora et al., 2011), focal lesions, and brain tumors.

Some limitations of the present study must be considered. Firstly, the involvement of a larger sample size for both groups of patients with dementia will be necessary. Specifically, in the group of patients with FTD, future studies should examine the impact of the different dementia variants on social abilities. Additionally, utilizing more and/or more refined tests than in the present study, with further tasks and tests, will enable a better understanding of the changes in the various components of ToM and emotion recognition in various forms of dementia.

Summing up, in the current study we found that FTD patients are significantly impaired in social cognition abilities, both in visual and in auditory modality, as compared to both AD patients and HC. On the other hand, in AD the emotional recognition impairment is prevalent in the auditory modality. Therefore, the introduction of the evaluation of these aspects in the clinical neuropsychological assessment could provide new insights into the cerebral localization of emotional and social skills, and into the neurodegenerative processes that may affect them.

Considering the clinical and social influence of social cognition impairments in these two neurodegenerative diseases, this study aimed to provide a more comprehensive characterization of their impact. Specifically, we employed an extensive assessment protocol designed to evaluate both visual and auditory processing across basic and social emotions within the same patient groups. While these tests may not produce clear differences sufficient for individual diagnosis, our objective is to offer a robust and reliable framework for delineating the behavioral and emotional profiles characteristic of AD and FTD. This, in turn, can serve as a valuable tool for enhancing our understanding of these diseases and facilitating improved clinical management strategies, including tailored therapeutic interventions and caregiver support.

## Data Availability

The raw data supporting the conclusions of this article will be made available by the authors, without undue reservation.
